# Altered gene expression of *VEGF*, *IGFs* and *H19* lncRNA and epigenetic profile of *H19*-DMR region in endometrial tissues of women with endometriosis

**DOI:** 10.1186/s12978-022-01406-w

**Published:** 2022-04-22

**Authors:** Sedigheh Kamrani, Elham Amirchaghmaghi, Firouzeh Ghaffari, Maryam Shahhoseini, Kamran Ghaedi

**Affiliations:** 1 Department of Cell and Molecular Biology and Microbiology, Faculty of Biological Science and Technology, University of Isfahan, Hezar Jerib Ave, Azadi Square, Isfahan, Iran; 2 Department of Endocrinology and Female Infertility, Reproductive Biomedicine Research Center, Royan Institute for Reproductive Biomedicine, ACECR, Tehran, Iran; 3 Department of Regenerative Biomedicine, Cell Science Research Center, Royan Institute for Stem Cell Biology and Technology, ACECR, Tehran, Iran; 4 Department of Genetics, Reproductive Biomedicine Research Center, Royan Institute for Reproductive Biomedicine, ACECR, P.O.Box: 19395-4644, Tehran, Iran; 5 Reproductive Epidemiology Research Center, Royan Institute for Reproductive Biomedicine, ACECR, Tehran, Iran; 6 Department of Cell and Molecular Biology, School of Biology, College of Science, University of Tehran, Tehran, Iran

**Keywords:** Endometriosis, Vascular endothelial growth factor, Insulin-like growth factors, H19 long noncoding RNA, Epigenetic

## Abstract

**Background:**

Endometriosis, as chronic estrogen-dependent disease, is defined by the presence of endometrial-like tissue outside the uterus. Proliferation of endometrial tissue and neoangiogenesis are critical factors in development of endometriosis. Hence, vascular endothelial growth factor (VEGF) as well as insulin‐like growth factor 1 and 2 (IGF1, 2) may be involved as inducers of cellular proliferation or neoangiogenesis. Imprinted long noncoding RNA H19 (lncRNA H19) has been suggested to be involved in pathogenesis of endometriosis via regulation of cellular proliferation and differentiation. Epigenetic aberrations appear to play an important role in its pathogenesis. The present study was designed to elucidate *VEGF, IGF1, IGF2* and *H19* lncRNA genes expression and epigenetic alterations of differentially methylated region (DMR) of *H19* (*H19*-DMR) regulatory region in endometrial tissues of patients with endometriosis, in comparison with control women.

**Methods:**

In this case–control study, 24 women with and without endometriosis were studied for the relative expression of *VEGF*, *IGF1*, *IGF2* and *H19* lncRNA genes using real-time polymerase chain reaction (PCR) technique. Occupancy of the MeCP2 on DMR region of *H19* gene was assessed using chromatin immunoprecipitation (ChIP), followed by real-time PCR.

**Results:**

Genes expression profile of *H19*, *IGF1* and *IGF2* was decreased in eutopic and ectopic endometrial tissues of endometriosis group, compared to the control tissues. Decreased expression of *H19* in ectopic samples was significant in comparison with the controls (P < 0.05). Gene expression of *VEGF* was increased in eutopic tissues of endometriosis group, compared to control group. Whereas its expression level was lower in ectopic lesions versus eutopic and control endometrial samples. ChIP analysis revealed significant and nearly significant hypomethylation of *H19*-DMR region II in eutopic and ectopic samples, compared to the control group respectively. This epigenetic change was aligned with expression of *IGF2*. While methylation of *H19*-DMR region I was not significantly different between the eutopic, ectopic and control endometrial samples.

**Conclusion:**

These data showed that *VEGF*, *IGF1*, *IGF2* and *H19* lncRNA genes expression and epigenetic alterations of *H19* lncRNA have dynamic role in the pathogenesis of endometriosis, specifically in the way that hypomethylation of *H19*-DMR region II can be involved in *IGF2* dysregulation in endometriosis.

## Plain language summary

Endometriosis as an estrogen-dependent chronic inflammatory disease is characterized by the growth of endometrial-like tissue outside the uterus. In this study, to evaluate the effect of genetic and epigenetic factors involved in this disease, *VEGF*, *IGF1*, *IGF2* and *H19* lncRNA genes expression and epigenetic alterations of *H19*-DMR regulatory region in endometrial tissues of 12 patients with endometriosis and 12 normal women (as control) were assessed. The results showed that expression of *H19*, *IGF1* and *IGF2* genes was decreased in eutopic and ectopic endometrial tissues of endometriosis group in comparison with control group. Expression of *VEGF* gene was increased in eutopic tissues of endometriosis group compared to the control group. Whereas its expression level was lower in ectopic lesions versus eutopic and control endometrial samples. Methylation of *H19*-DMR region II was decreased in eutopic and ectopic samples compared to the control group. This epigenetic change was aligned with *IGF2* expression. Methylation of *H19*-DMR region I was not significantly different between eutopic, ectopic and control endometrial samples. Findings of this study showed that *VEGF*, *IGF1*, *IGF2* and *H19* lncRNA genes expression and epigenetic alterations of *H19* lncRNA have dynamic role in the pathogenesis of endometriosis. Additionally, hypomethylation of *H19*-DMR region II may be involved in impaired *IGF2* regulation in endometriosis.

## Background

Endometriosis is an estrogen-dependent gynecological disease in women of child-bearing age. This chronic disease is defined as presence of endometrial glands and stromal cells outside the uterine cavity [1]. Endometriosis affects 6–10% of women in reproductive-age. Endometriotic lesions are often found in the ovaries, fallopian tubes and peritoneal cavity [2]. Pelvic pain and infertility are common symptoms of endometriosis. Other symptoms of endometriosis include dysmenorrhea, irregular menstruation, dyspareunia and dysuria [3]. Endometriosis is considered as a multifactorial disease affected by genetic, hormonal, immunological and environmental factors [4]. On the basis of previous studies, adhesion, proliferation of endometrial cells, cellular invasion and neoangiogenesis are key factors in the pathogenesis of endometriosis [5]. Therefore, growth factors including insulin-like growth factors (IGFs) may play roles in inducing of cellular proliferation and differentiation [6]. Endometrial tissue produces IGF1 and IGF2, playing important roles in growth and differentiation of endometrial cells [7]. It has been shown that IGF1 prevents apoptosis and acts as a mitogenic factor for endometrial cells [8]. In addition, it has been reported that IGF-1 protein concentration is increased in the peritoneal fluid of patients with endometriosis compared to control women [9]. *IGF2* gene is imprinted and paternally expressed in prenatal mammalian tissues [10]. It has been shown that IGF1 deficiency causes infertility and hypoplasia of uterus in female mice, and it was suggested that IGF1 has main role in uterine growth and function [6]. Human endometrial epithelial and stromal cells express *IGF1* and *IGF2* that their highest expression levels are in the late and early proliferative phase, respectively [11]. It has been suggested that angiogenesis is necessary for growth and survival of endometriotic lesions [12]. Vascular endothelial growth factor (VEGF) is the most important angiogenesis factor that causes endothelial proliferation, vasodilation and increases vascular permeability [13]. Several researches showed that level of VEGF mRNA is higher than control group, in endometriosis, which indicated the main role of *VEGF* in angiogenesis related to endometriosis [14]. Long noncoding RNAs (lncRNAs) are non-protein-coding transcripts with longer than 200 nucleotides. This type of RNAs are involved in important functions of various biological processes in cancer [15]. H19, as lncRNA, is located in an imprinting region on chromosome 11p15.5 of human. It plays a major role in embryonic development and regulating growth. *H19* is expressed from maternally allele [16]. Structurally, *H19* gene includes five exons and four introns that produce a 2.3-kb lncRNA after splicing [17]. In the human endometrium, expression of H19 increases at the late proliferative phase [18]. In recent years, studies suggested that endometriosis can be considered as an epigenetic disease which involves DNA modifications. Epigenetics is composed of heritable phenotype changes which are not caused by alterations in the DNA sequence. Evidences suggested that various epigenetic modifications, such as DNA methylation, may play a main role in the etiology of endometriosis [19]. DNA methylation generally acts to suppress gene transcription which occurs through binding methylated DNA-binding proteins, such as methyl CpG binding protein 2 (MeCP2), to methyl-CpG sites [20]. *H19* and *IGF2* are reciprocally imprinted genes. *IGF2* gene is located 90 kb away from *H19* gene. Expression of these two genes is coordinately regulated through an intergenic differentially methylated region (DMR) and downstream enhancers. DMR region, also called imprinting control region (ICR), are located in 2–4 kb upstream of the *H19* transcription site. So that, *H19* is expressed from maternal allele while *IGF2* is expressed from the paternal allele [21].The responsible mechanism for controlling *H19* and *IGF2* imprinting consists of binding MeCP2 or CCCTC-binding factor (CTCF) based on the methylation status of DMR. This region includes seven binding sites for insulating factor CTCF, which methylation changes in the sixth CTCF-binding site is related to the altered expression of *H19* and *IGF2* genes in diseases [22]. On the paternal chromosome, binding MeCP2 to the hypermethylated DMR causes gene expression of *IGF2* and repression of *H19*. Conversely, on the maternal chromosome, *H19* is expressed and *IGF2* is repressed by CTCF protein binding to the hypomethylated DMR [23]. H19 regulates gene expression through epigenetic mechanisms by interacting with chromatin-modifying complexes. Therefore, lncRNA H19 could play role in the pathogenesis of some diseases [24]. The role of *H19* was showed in infertility related diseases. Korucuoglu et al*.* showed that *H19* expression is reduced in endometrial tissues of unexplained infertile women [25], while Ghazal et al*.* revealed that H19 regulated endometrial tissue proliferation by altering IGF signaling in endometriosis [26]. In the present study, we evaluated and compared expression levels of *H19* RNA factor and angiogenic (*VEGF*) and proliferative (*IGF1*, *IGF2*) genes in endometrium of non-endometriosis women in comparison with eutopic and ectopic tissues of endometriosis women. Additionally, we investigated modifications of DNA methylation (MeCP2 incorporation) of the *H19*-DMR regulatory region in these tissues.

## Methods

### Subjects and tissue samples

In this case control study, 24 women undergoing diagnostic laparoscopy at Royan Institute, (Tehran, Iran) were enrolled from 2019 to 2020. According to diagnostic laparoscopy findings, 12 women with endometriosis and 12 women without endometriosis were respectively considered as endometriosis and control groups. All women were 20–45 years old and they had no endometrial hyperplasia or neoplasia. In addition, none of them did receive hormonal drugs within the last three months. Women without endometriosis in diagnostic laparoscopy considered as control group. They had no evidence of pathologic uterine disorder. All of the enrolled patients suffering endometriosis were in the stage III or IV of disease, according to the revised American Society for Reproductive Medicine (rASRM) classification [27]. All endometrial samples were collected during proliferative phase of menstrual cycle. Control and eutopic endometrial samples (12 control and 12 eutopic samples) were obtained using pipelle. Ectopic samples (12 ectopic tissues) were collected during laparoscopy. After collection of endometrial samples (total 36 samples), the tissues were immediately transferred into two separate cryovials. One cryovial containing RNA later solution used to study gene expression and the other without RNA later solution for epigenetic evaluations. All samples were stored at − 80 °C until performing the analysis.

This study was approved by the Institutional Ethics Committee of Royan Institute (code: IR.ACECR.ROYAN.REC.1398.95). All enrolled women signed the informed consent form before tissue samples collection.

### RNA extraction and cDNA synthesis

Endometrial tissues were removed from RNA later and then homogenized in 1 ml TRIzol reagent (Kiazol, Iran). Total RNA extraction was done using TRI reagent protocol according to the manufacturer instruction. It was subsequently treated with DNaseI (Takara, Japan) to remove genomic DNA contamination. Concentration and purity of RNA samples were assessed by Nanodrop 2000 spectrophotometer (Thermo Scientific, USA). Complementary DNA (cDNA) synthesis was performed using TaqMan reverse transcription kit (Takara, Japan), according to the manufacturer’s instruction.

### Quantitative real time polymerase chain reaction (qRT-PCR)

Gene expression assessment was carried out by quantitative real time polymerase chain reaction (qRT-PCR) using the Step-One RT-PCR system (AB Applied Biosystems, USA) and with the primers designed for *VEGF, IGF1, IGF2, H19* (the primers listed in Table 1). Human glyceraldehyde dehydrogenase (*GAPDH*) was used as housekeeping gene. Gene expressions were calculated according to the 2^−ΔΔCT^ algorithm, by normalizing their expression to the *GAPDH*, as an internal standard.

**Table 1:** Primer pairs used in this study

Genes	Primer Sequences (5′–3′)	Product length (bp)
A. Real-time RT-PCR primers		
VEGF	F: ACCCACCCACATACATAC	151
	R: CAGCAGTCAAATACATCCAG	
IGF1	F: ATGCTCTTCAGTTCGTGTG	148
	R: CAATACATCTCCAGCCTCCT	
IGF2	F: CCTCTATCCTTGATACAACAGC	121
	R: AATTCGTCTGATTGTCCAGG	
H19	F: GTGACAAGCAGGACATGAC	121
	R: GAAGTAAAGAAACAGACCCGC	
GAPDH	F: CTCATTTCCTGGTATGACAACGA	122
	R: CTTCCTCTTGTGCTCTTGCT	
B. Real-time ChIP PCR primers		
H19-DMR	F: CTACAACCAATTCCGTGCCA	127
	R: TAGTGTGAAACCCTTCTCGC	
H19-DMR	F: TATCTCAGCCAACACAAGGA	127
	R: GCCAGACATTAACATTCCCA	

F and R are referred as forward and reverse primers respectively

### Chromatin immunoprecipitation (ChIP) assay

Chromatin immunoprecipitation (ChIP) experiments were performed by using the Orange ChIP kit (Diagenode, Belgium), according to the manufacturer’s instructions. Briefly, cross-linking between DNA and protein was fixed in homogenized endometrial tissues by adding formaldehyde (37%; Sigma, USA). Next, by adding glycine, the cross-linking reaction induced by formaldehyde was quenched. Sonication was used to fragment chromatin to an average DNA fragment size of 200–600 bp using the Bioruptor Sonication System (Diagenode, UCD 200 Bioruptor). One percent of the sheared chromatin was saved, as control input DNA (without adding any antibody), while the rest was incubated with anti-MeCP2 antibody (Abcam, UK) for immunoprecipitation. Real time quantitative PCR (qPCR) was used to analyze level of DNA methylation modifications of *H19-*DMR region with specific primer sets (Table 1). The primers were designed to amplify two different regions of *H19*-DMR. Data is reported based on the fold enrichment of different immunoprecipitated DNA relative to 1/100 dilution of input chromatin. The % input was determined using the following formula: % input = 2^(CtP inputP – CtP ChIPP)^ B B × Fd × 100%.

### Statistical analysis

Data analyses were performed using SPSS 16 software. One-way analysis of variance (ANOVA) test and non-parametric Kruskal–Wallis test were used when distribution of the values was normal and not normal, respectively. Values were expressed as mean ± standard error of mean (SEM). A *p*-value less than 0.05 was taken into consideration as statistical significant.

## Results

### Expression analysis of *VEGF, IGF1, IGF2* and *H19* genes

**Fig. 1:**
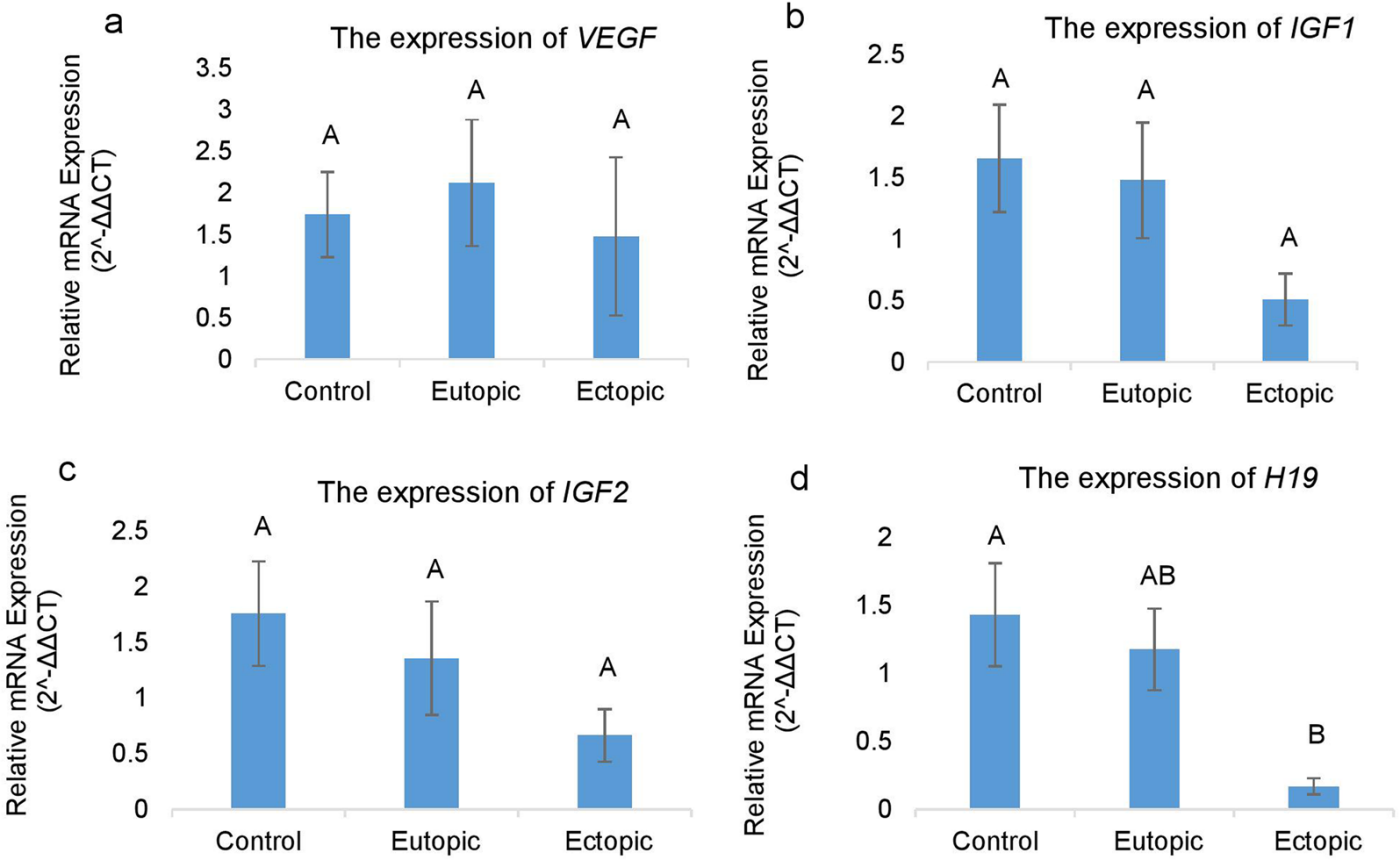
Relative mRNA expression levels of *VEGF, IGF1, IGF2* and *H19* genes in eutopic and ectopic tissue samples (*n* = 12 in each group) from endometriosis patients vs. control group (*n* = 12), in proliferative phase. The results are expressed as 2^−ΔΔCT^ (mean ± SEM). Expression of *H19* significantly decreased in ectopic samples compared to control group. Different letters indicate significant difference between the eutopic, ectopic and control groups in *p* < 0.05

To determine relative expression levels of the *VEGF, IGF1, IGF2* and *H19* genes in eutopic, ectopic and control groups, qRT-PCR was performed during proliferative phase of menstrual cycle. According to Fig. 1A, gene expression level of *VEGF* was increased in eutopic tissue, in comparison with control and ectopic samples. There was a decrease in *VEGF* level in ectopic endometrium versus eutopic samples and control group. Although these differences were not statistically significant (*p* > 0.05; Fig. 1A). Expression levels of *IGF1* and* IGF2* were decreased in ectopic endometriotic lesions, compared to the eutopic and control groups. In addition, expression levels of these two genes were lower in eutopic samples compared to control group, although the differences were not significant (*p* > 0.05; Fig. 1B and C). As shown in Fig. 1D, expression of *H19* was decreased in eutopic and ectopic endometrial lesions of endometriosis group, in comparison with the control group. Statistical analyses showed that decrease in ectopic samples was significant compared to control group, while it was marginally significant compared to the eutopic samples (*p* = 0.01 and *p* = 0.056, respectively). However, there was no significant difference in expression profile of *H19* between eutopic and control groups (*p* > 0.05; Fig. 1D).

### DNA methylation of the DMR region of *H19* gene

**Fig. 2:**
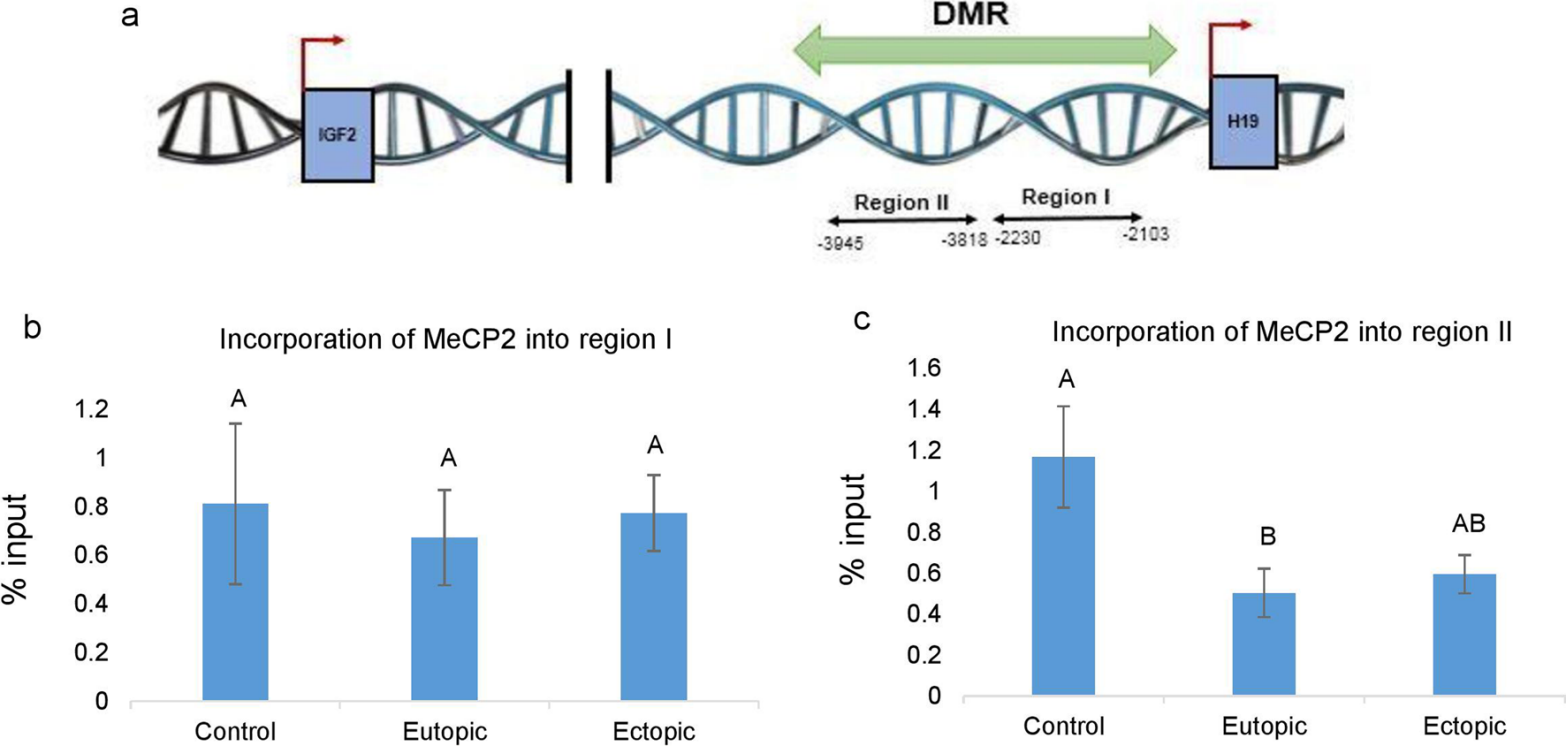
DNA methylation profile of *H19-*DMR regulatory regions by chromatin immunoprecipitation (ChIP) assay. **A** The regions amplified by qPCR are shown by arrows, and nucleotide numbers are relative to the transcription start site. Different letters indicate significant differences between the groups (*p* < 0.05). Incorporation of MeCP2 on the regulatory regions of **B** region I*,* **C** region II in endometriosis and control groups. The data showed significant hypo-methylation in region II in eutopic group compared to control group. There was no significant difference in the incorporation of MeCP2 in region I between the eutopic, ectopic and control samples. The results are expressed relative to a 1/100 dilution of input chromatin (mean ± SEM)

In order to evaluate DNA methylation on DMR of *H19*, MeCP2 incorporation on this region was investigated in eutopic, ectopic and normal tissues by ChIP assay. Two sub-regions (region I and region II in Fig. 2A) were analyzed within *H19*-DMR. Region I contains the sixth CTCF-binding site. It has been proposed that this site has a key regulatory domain for the imprinted expression of *H19* and *IGF2* genes [28]. ChIP analysis revealed that MeCP2 incorporation at the first DMR region of *H19* gene was not significantly different between the eutopic, ectopic and control samples (*p* > 0.05; Fig. 2A). Furthermore, a significant hypomethylation at the second DMR region of *H19* gene was observed in the eutopic samples compared to the control group (*p* = 0.02), while this decrease was marginally significant in the ectopic samples versus control samples (*p* = 0.056). However, there was no significant difference in DNA methylation (MeCP2 incorporation) of the second region of *H19*-DMR, between the ectopic and eutopic endometrial samples (*p* > 0.05; Fig. 2B).

## Discussion

Despite many studies, the exact cause of endometriosis is yet unclear. Currently, several evidences showed that neo-angiogenesis was a major element in implantation and survival of endometrial lesions outside the uterine cavity**.** So, VEGF may have important roles in the pathogenesis of endometriosis [29, 30]. In the present study, gene expression of *VEGF* was increased in the eutopic endometrial tissues of women with endometriosis compared to the controls. Moreover, expression of this gene was lower in the ectopic lesions versus eutopic and control endometrial samples. This finding was consistent with Cosín et al. study showing that mRNA expression of *VEGF* was increased in the eutopic endometrium of endometriosis compared to the controls in proliferative phase [31]. Rashidi et al*.* showed that there was no significant difference in *VEGF* mRNA expression between endometriosis and control groups. However, it was reported that mRNA expression of *VEGF* in the case group was higher in the secretory phase than in the proliferative phase [32]. In 2018, Zhang et al*.* showed that expression of *VEGF* was significantly increased in both serum and endometrial tissues and it is correlated with the R-AFS (American Fertility Society classification) stage [33]. In relation to endometriotic lesions, Takearaet et al*.* indicated that expression of *VEGF* mRNA was increased in the endometriotic tissue (early stages) compared to eutopic endometrium [34]. However, in the present study, we did not detect any increase in *VEGF* mRNA expression in ovarian endometriomas (stages III–IV). In agreement with our study, a reduction of *VEGF* level was reported in ovarian endometrioma compared to the eutopic endometrium in endometriosis [35, 36]. Additionally, decreased angiogenic activity of endometriotic lesions was observed in the advanced stages [37, 38]. In addition, Bourlev et al. showed that in ectopic endometrium, samples with high proliferative activity in glandular epithelial cells indicated greater expression of *VEGF-A* in stromal and epithelial cells than samples with low proliferative activity [39]. Results suggested that overexpression of *VEGF* in the eutopic endometrium of endometriosis could represent higher angiogenic activity, which might contribute to the increased ability of endometrial cell implantation at the ectopic sites. Insulin-like growth factor (IGF) axis appears to be involved in regulation of endometrial cell proliferation and differentiation [40]. LncRNA H19 could regulate IGF‐1 signaling pathway and consequently proliferation as well as apoptosis of endometrial stromal cells [41]. Previous studies reported close relationship between H19 and IGF1 signaling pathway. H19 can also recruit epigenetic modifiers, acting as a guide to suppress gene expression [42, 43]. Our results showed that women with endometriosis had decreased *IGF1* expression as well as decrease in the level of *IGF2* expression in the eutopic and ectopic endometrium of endometriosis group compared to the controls. In agreement with our study, Sbracia et al. showed decreased *IGF2* expression level in the eutopic and ectopic epithelial endometrial cells of endometriotic in comparison with control. In addition, *IGF1* was downregulated in the eutopic endometrium of women with endometriosis compared to the control endometrial samples, whereas increased *IGF1* expression in fibrotic peritoneal adhesions was observed [40]. Milingos et al. reported that a significant decrease in *IGF1* expression in endometriotic cyst in comparison to eutopic endometrium of women with endometriosis [44]. Our results were inconsistent with Arablou et al. study demonstrating significantly higher *IGF1* expression in the ectopic endometrial stromal cells (EESCs) compared to EuESCs and CESCs [45]. This apparent difference is probably due to the cell culture. Because in vitro manipulation of isolated stromal cells and lack of in vivo crosstalk with other cells may play a role in the altered expression of *IGF1* in cell culture.

According to the present results, *H19* gene expression was significantly lower in the ectopic lesion compared to the eutopic and control endometrium. Similar to our results, Ghazal et al. detected that *H19* expression was decreased in the eutopic endometrium of patients with endometriosis in comparison with the controls [26]. Furthermore, in 2019 Liu et al. showed that lncRNA H19 was downregulated in mononuclear cells from peritoneal fluid (PFMCs) of patients with endometriosis [46]. But a few studies exhibited that lncRNA H19 expression in the ectopic and eutopic endometrial tissues of endometriosis was significantly higher than the normal endometrium [47–49]. This discrepancy is likely the result of differences in race and maybe stage of disease. So, more genetic investigations are needed. In addition our results were based on in vivo experiments, so further in vitro studies are required.

In the present study, decreased expression of *IGF1* could be due to decreased *H19* expression level. It was determined that IGF1, mediated via IGF1 receptor (IGF1R), activated PI3K/AKT and Ras/Raf/MAPK signal transduction pathways, which enhanced uterine cell proliferation [7]. During proliferative phase of the menstrual cycle, estradiol induces *H19* expression in the endometrium. Uprising of H19 causes increased level of Igf1r protein. This increase leads to upregulating IGF1 signaling with an increased proliferation of endometrial stromal cells [26]. This pathway seems to be changed in women with endometriosis. Therefore, in our study, decreased *H19* expression probably reduces expression of *IGF1* and thus decreased IGF1 signaling, leading to reduced stromal cell proliferation rate in endometriosis patients. These alterations in proliferation of endometrial stromal cell may be a potential mechanism for infertility in women with endometriosis. Since endometrioma indicates late stages of endometriosis, reduction of *IGF1* expression in cysts is likely related to the disease status. In addition, *H19* expression level may be correlated to disease progression and infertility.

In our study, decreased expression of *IGF2* could be due to two reasons; epigenetic mechanisms by H19 and reducing methylation in *H19/*ICR. Earlier studies showed that lncRNA H19 binds methyl-CpG-binding domain protein 1 (MBD1). LncRNA H19–MBD1 complex binds to methylated DNA after which recruits histone lysine methyltransferases (KMTs) to silence these genes via chromatin compaction (H3K9 methylation) [50]. The function of *H19*-DMR methylation on *IGF2* expression, resulting from loss of imprinting of IGF2, was observed in many studies [51, 52]. As previously mentioned, DNA methylation in *H19-*DMR region is epigenetic alterations which can induce imprinting and affect gene expression. To determine whether reduced *IGF2* and *H19* gene expressions in endometriosis was because of epigenetic modifications or not, we then analyzed epigenetic alterations of the *H19-*DMR regulatory regions. Generally, in normal conditions when the ICR is methylated, MeCP2, as part of the methyl-binding protein family (MBDs), binds to methylated CpG dinucleotides. This is the main mechanism through which DNA methylation can suppress transcription of *H19* gene and the enhancer region is capable of interacting with *IGF2* to promote expression. Conversely, when ICR is not methylated, CTCF binds and allows the same enhancer region to promote *H19* expression. Therefore, this region and its methylation are essential for both *H19* and *IGF2* expression [53, 54].

Our results showed that methylation modifications on the regulatory regions of *H19*-DMR in region II is consistent with the pattern of *IGF2* gene expression. However, expression level of *H19* was not associated with methylation changes in regions I and II. Furthermore, incorporation of MeCP2 in region I within DMR region of *H19* gene was not significantly different between endometriosis and normal tissues. It has been shown that hypermethylation of CTCF-binding site at the *H19*/ICR increased expression of *IGF2* in ovarian cancer [55]. Alternatively, in esophageal squamous cell carcinoma, *H19* CTCF-binding site 6 (CBS6) hyper-methylation correlates with overexpression of *IGF2* in patients [56]. In another study, it was shown that alteration of *H19/IGF2* expression patterns, due to hypo-methylation of *H19*-DMR, may play roles in the pathogenesis of pregnancy complications [57]. However, no study was performed on methylation modifications of *H19*-DMR in endometriosis. Results of the current study showed that methylation modifications of region II of *H19*-DMR can alter expression level of *IGF2*, but it has no effect on *H19* expression. It is hypothesized that epigenetic modifications of the other regions of *H19*-DMR can probably affect expression profile of *H19* gene.

## Conclusion

In conclusion, according to the mentioned results, overexpression of *VEGF* in the eutopic endometrium could lead to implantation of endometrial fragments in extra uterine cavity**.** Decreased *H19* expression in endometriosis lesions probably decreased *IGF1* and *IGF*2 expression. This pattern implies that the cells of endometriotic tissue possibly undergo an impairment of cellular growth regulation and differentiation. Finally, epigenetic findings of the present study showed that region II of *H19*-DMR can affect expression profile of *IGF2*, although more investigations are required to clarify roles of the further epigenetic modifications in this region as well as the studying more target genes for *H19*.

## Data Availability

Supporting and raw data are available upon a reasonable request to the corresponding author.

## Abbreviations

VEGF: Vascular endothelial growth factor
IGF1: Insulin‐like growth factor 1
IGF2: Insulin‐like growth factor 2
lncRNAs: Long noncoding RNAs
MeCP2: Methyl CpG binding protein 2
DMR: Differentially methylated region
ICR: Imprinting control region
ChIP: Chromatin immunoprecipitation
